# Mimicking the Impact of Infant Tongue Peristalsis on Behavior of Solid Oral Dosage Forms Administered During Breastfeeding

**DOI:** 10.1016/j.xphs.2016.08.006

**Published:** 2017-01

**Authors:** Rebekah L. Scheuerle, Richard A. Kendall, Catherine Tuleu, Nigel K.H. Slater, Stephen E. Gerrard

**Affiliations:** 1 Department of Chemical Engineering and Biotechnology, BioScience Engineering Research Group, University of Cambridge, New Museums Site, Cambridge, UK; 2 Department of Pharmaceutics, School of Pharmacy, University College London, London, UK

**Keywords:** *in vitro* models, oral drug delivery, pediatric, physical characterization, drug delivery systems, API, active pharmaceutical ingredient, NSDS, nipple shield delivery system, PEEK, polyetheretherketone, SB, sulforhodamine B, TM, tongue mimic, TMS, tongue mimic system

## Abstract

An *in vitro* simulation system was developed to study the effect of an infant's peristaltic tongue motion during breastfeeding on oral rapidly disintegrating tablets in the mouth, for use in rapid product candidate screening. These tablets are being designed for use inside a modified nipple shield worn by a mother during breastfeeding, a proposed novel platform technology to administer drugs and nutrients to breastfeeding infants. In this study, the release of a model compound, sulforhodamine B, from tablet formulations was studied under physiologically relevant forces induced by compression and rotation of a tongue mimic. The release profiles of the sulforhodamine B in flowing deionized water were found to be statistically different using 2-way ANOVA with matching, when tongue mimic rotation was introduced for 2 compression levels representing 2 tongue strengths (*p* = 0.0013 and *p* < 0.0001 for the lower and higher compression settings, respectively). Compression level was found to be a significant factor for increasing model compound release at rotational rates representing nonnutritive breastfeeding (*p* = 0.0162). This novel apparatus is the first to simulate the motion and pressures applied by the tongue and could be used in future infant oral product development.

## Introduction

Breastfeeding is a complex, variable process dependent on cultural practices, mother and child behaviors, and physiology. In general, breastfeeding begins with milk expression by a milk-ejection reflex, which is then followed by a nutritive breastfeeding phase, followed by a nonnutritive phase.^1^, ^2^

During the nutritive sucking phase, the bulk of the feed volume is delivered, with up to 90% of the feed volume delivered in the first 4 min.^1^ Breastfeeding then continues at a reduced average flow rate and is nonnutritive, with the infant mainly feeding for comfort.^1^ The infant's behavior varies between nutritive and nonnutritive breastfeeding, but in both cases, motion of the tongue is coordinated with creation of an intraoral vacuum formed by the orientation of the infant's mouth.^3^, ^4^, ^5^ Peristaltic motion of the tongue has been documented by Niikawa et al.^6^ who observed phase differences in pressure measurements taken *in vivo* using an artificial nipple loaded with force sensors.

The infant tongue is thought by some to be influential in milk expression, due to a possible role in stripping the nipple of milk via a peristaltic action.^5^, ^1^ The initiation of the peristaltic motion by the upward movement of the tip of the tongue is accompanied by compression from the lower jaw.^5^ At the end of the peristaltic tongue motion, the jaw drops back down with the tongue, and a negative intraoral vacuum then occurs.^5^ The importance of the tongue motion to milk flow rates is contested though, with some studies supporting that vacuum pressure during breastfeeding predominately influences milk flow.^3^

**Figure 1:**
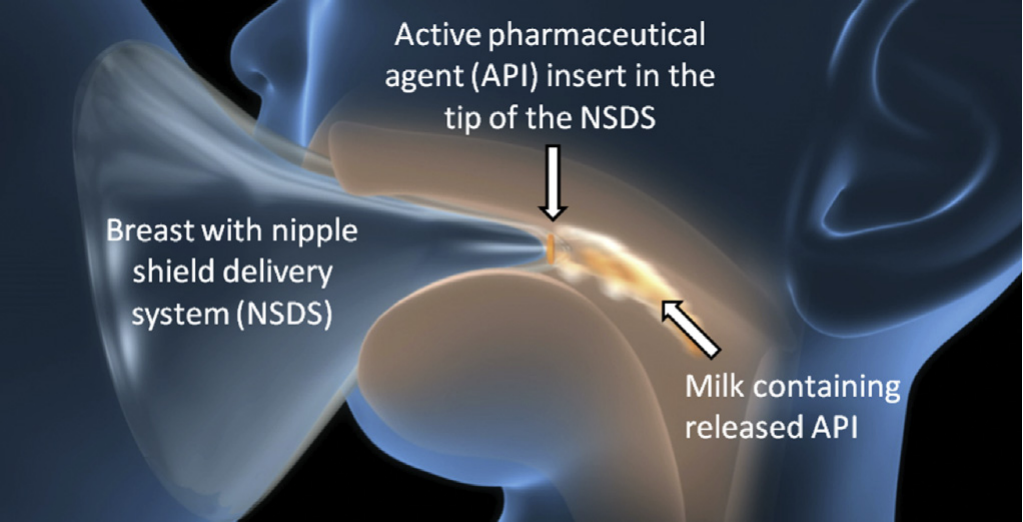
Nipple shield delivery system illustration.www.justmilk.org.

Gerrard et al.^7^ have built a breastfeeding simulation apparatus for mimicking the suction component of the breastfeeding process. In this apparatus, the mother's lactation and infant's suction behaviors are mimicked but not the tongue action of the infant. The apparatus has been used in feasibility testing for the nipple shield delivery system (NSDS), a novel infant drug delivery device under development for delivering therapeutics or nutrients to breastfeeding infants.^8^, ^7^, ^9^, ^10^ The silicone device, as shown in Figure 1, holds an insert containing active pharmaceutical ingredient(s) (API). When worn by a mother during breastfeeding, API is released into milk consumed by the infant. Using the breastfeeding simulation apparatus, Gerrard et al.^7^ have tested the device under various physiologically relevant conditions, including under the influence of a range of human milk flow rates expected during maternal lactation in combination with the influence of suction pressures representative of those induced by the infant during breastfeeding.

The apparatus focused on the suction-based impacts of breastfeeding on drug release. It is hypothesized that infant tongue movement during breastfeeding could also impact API release from the NSDS by increasing the rate of tablet disintegration due to pressures exerted by the peristaltically moving tongue during breastfeeding.

To characterize the impact of peristaltic tongue motion during breastfeeding on the release of model compounds from tablets to be used in the NSDS, a tongue mimic system (TMS) was designed and constructed. Tongue mimics have been reported in the literature^11^ for the study of swallowing. To the authors' knowledge, the present research describes the first system which mimics infant tongue motion specifically during breastfeeding.

A form of the TMS would be a useful addition to the breastfeeding simulation apparatus used by Gerrard et al.^7^ for improved *in vitro* simulation of the breastfeeding process. Through simulation of the peristaltic motion of an infant tongue, a process that may impact milk flow behavior through the NSDS and tablet disintegration in the NSDS improved NSDS feasibility studies could be possible. This system could also simulate the compression forces on the NSDS when positioned in the pharyngeal space resulting from tongue and jaw motion during breastfeeding.^12^

Furthermore, the tongue mimic system could be useful as a biorelevant tool in characterizing chewable tablets, soft chews, or boiled sweets. A system like this could be useful in mimicking chewing, and therefore characterization of the effects of chewing on modified release solid dosage forms. If further developed and validated, this could potentially be a useful supplement to other forms of tablet disintegration testing^13^, ^14^, ^15^ and texture analysis disintegration testing.^16^

## Materials

### Tablets

Model tablets based on conventional rapidly disintegrating tablet formulations were manufactured. Tablets were formulated with sulforhodamine B (SB) (Sigma-Aldrich, Dorset, UK), a highly water soluble dye. It served as a model compound for potential APIs and could be quantified easily using a spectroscopic assay. During formulation, the tablet components including lactose as a filler, SB, and superdisintegrants sodium starch glycolate and croscarmellose sodium were blended based on standard tablet laboratory-scale manufacturing practice. The lubricant was then added, and the mixture was sieved at 500 μm before the final blending took place. A Manesty F3 Tablet Press (Liverpool, UK) with a biconvex 80 single punch and die set with an 8-mm diameter (Holland, Nottingham, UK) was used to directly compress the tablets to a target weight of 330 mg.

## Methods

### Characterization of Tablets

The tablets were characterized using standard United States Pharmacopeia methods for friability using a FR10000 Copley Friabilator (Nottingham, UK), and hardness using an Erweka TBH200 hardness tester (Heusenstamm, Germany). A Copley ZT 34 (Nottingham, UK) disintegration apparatus with a basket rack assembly was used for disintegration testing, with each tablet individually tested for disintegration time.

### Design of Tongue Mimic System

A novel TMS was designed to characterize release of a model API from rapidly disintegrating tablets in flowing fluid when exposed to pressures and peristaltic motion within the range of that induced by an infant tongue during breastfeeding.

The TMS included a reservoir for the fluid source, for which deionized water was chosen because of its ease to quantify the model compound in, homogeneity, and frequency of use in standard disintegration tests^13^ compared to human milk. Human milk is highly variable in composition, with protein, fat, and carbohydrate amounts varying between mothers and feeds.^17^ Future tests could use human milk but would require careful matching of milk composition to ensure reproducibility of results because media composition has been shown in some studies to influence tablet disintegration.^16^, ^18^

**Figure 2:**
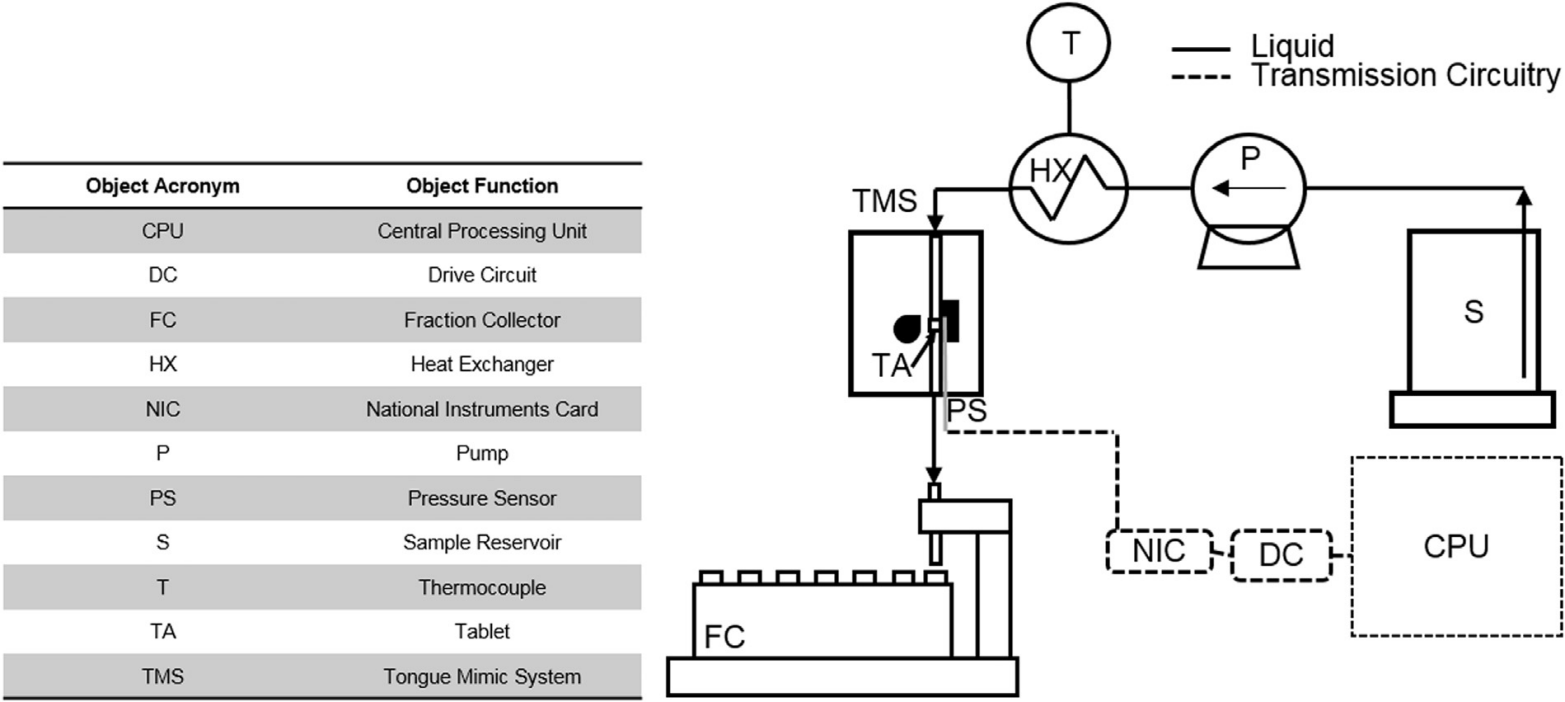
Process flow diagram of the tongue mimic system experimental apparatus.

The TMS also included a heat exchanger constructed from a Gallenkamp hot plate to bring the fluid to a physiologically relevant temperature, a Masterflex peristaltic pump (Cole-Parmer, UK) to pump the fluid from the reservoir, a purpose-built tongue mimic (TM) to hold a tube loaded with the tablet, followed by a SuperFrac fraction collector (GE Healthcare Sciences, Buckinghamshire, UK). A process flow diagram of the TMS is shown in Figure 2.

**Figure 3:**
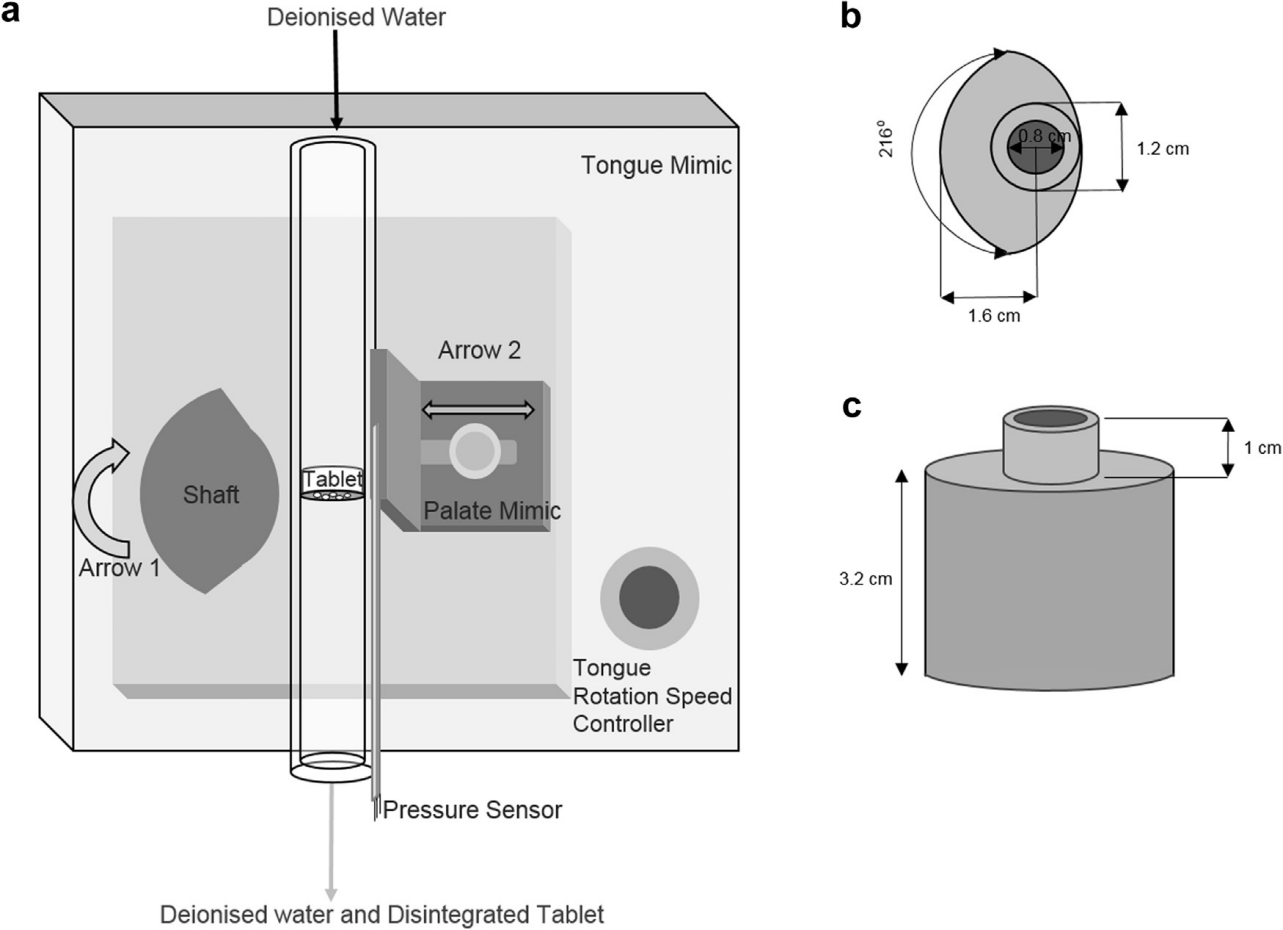
Illustration of the tongue mimic (a) where arrow 1 represents rotation which the tongue mimic is capable of and arrow 2 represents the plausible movement of the palate mimic for adjusting the amount of compression exposed to the tablet; dimensions of the tongue mimic shaft used in the TMS shown from the top (a) and side (b).

The TM as shown in Figure 3 was a modified Masterflex peristaltic pump (Cole-Parmer, UK) with the pump head replaced by a CAD-aided 3D-printed acrylonitrile butadiene styrene bit connected to a shaft to represent the tongue. The shaft's rotation rate was altered directly through varying the pump speed. A movable metal plate, chosen for durability, was affixed to the pump to mimic the hard palate of an infant's mouth.

The portion of the shaft representing the tongue was designed using AutoCAD (Autodesk, San Rafael, California) to include one nodule shaped such that a period of contact and period of no contact with the tablet-containing tubing would occur during each shaft rotation. This was designed to mimic the repeated contact and release of the nipple during breastfeeding.^5^ Because the suction portion of an infant suction cycle lasts for half of the cycle^19^ regardless of whether the infant is engaging in nutritive or nonnutritive breastfeeding,^5^ the shaft was designed to apply pressure to the sensor-containing region of the system for 50% of the rotation cycle.

The tube loaded with the tablet in the TM was placed between the shaft and the palate mimic. The tablet was held in place by a metal grate, chosen for its durability against deformation under pressure, which had 7 evenly placed 2-mm holes, similar in size to those present in the nipple shield delivery system.

The pressure transmits with dampening from the shaft through the tubing and tablet and is then measured by a Flexiforce Tekscan piezoresistive sensor (South Boston, MA, rise time 0.1–0.3 s, response time <5 μs). To calculate the percentage of the rotation cycle for which the shaft could apply pressure through the tubing and tablet to the pressure sensor, the number of degrees over which contact between the 2 was calculated out of those of a full cycle. This sensor location was chosen to minimize damage to and ensure repeatable placement of the sensor.

The sensor is connected to a drive circuit, which is connected to a cDAQ NI USB-6008 (National Instruments, Austin, TX) for data acquisition. Digital data from the cDAQ NI USB-6008 is fed into a computer where it is monitored and recorded using LabVIEW (National Instruments).

To calibrate the pressure sensor, known weight standards in the dynamic testing range to be used in experimentation were applied to the sensor, for which the pressure was calculated based on the standard mass, the acceleration of gravity, and the area of the sensor. The generated analog voltage data were collected using the cDAQ NI USB-6008. The digital data were processed by LabView at an acquisition rate of 1000 Hz. The voltage data were converted into resistance values followed by conductance values. Linear regression was used to define calibration curves for pressure as a function of conductance for different contact times.

### Experimental Method

The TMS is used to characterize release of SB from tablets in deionized water. Water was heated in the heat exchanger, resulting in a temperature range of 33.5°C-35.5°C to represent in the tablet-containing silicone tubing (inner diameter: 8 mm, outer diameter: 12 mm; Granta Pneumatics & Automation, Cambridge, UK) plausible mouth temperatures during use of the NSDS. Separate experiments were carried out at 2 levels of compression exerted on the tablet-containing tubing based on replicating physiologically relevant pressures exerted by structures in the infant mouth during breastfeeding. The compression amount was dictated by the distance of the tongue palate mimic from the tongue mimic's shaft.

Suction frequency is variable, ranging from 40-120 suction pulses/min, and depends largely on whether the infant is engaging in nutritive or nonnutritive breastfeeding averaging 1 suction cycles per second and 2 suction cycles per second respectively.^5^ To mimic multiple potential suction rates, for each compression setting, experiments were run with the tongue mimic's shaft rotational rate set to speeds of 58.5 ± 0.3 (R1) or 105.4 ± 0.5 RPM (R2). A set of experiments corresponding to 0 RPM and constant pressure was also performed as a control. During these studies, the TMS was turned on just long enough for the raised portion of the TM's shaft to reach contact with the tablet-containing portion of the tubing, after which rotation was ceased. A set of experiments with no applied pressure and no rotation was also performed, as a control.

Reviews of reported milk flow during breastfeeding have indicated it is highly variable with rates varying from 0.4-16.8 mL/min.^7^ Therefore, the TMS was set to a flow rate of 4.92-4.97 mL/min to fall in this range.

To characterize the compression settings of the TMS, a nondisintegrating polyetheretherketone (PEEK) tablet, the dimensions of an SB tablet, was placed in the apparatus. The resulting pressures measured by the sensor at the 2 tested rotational rates were recorded when no flowing water was pumped through the TMS. The resultant peak pressures for the first 10 cycles at each setting are shown in Table 1 for the purposes of documenting the compression applied by the system. The PEEK tablets allowed this characterization without introducing variability due to fracturing possible with the therapeutic model tablets.

### Detection of Sulforhodamine B in Water

Quantification of SB release was performed using a Spectrostar Nano spectrophotometer (Ortenberg, Germany). A calibration curve was used to determine the amount of SB present in diluted, vortexed samples that exited the TMS based on the absorbance measured at a maximum wavelength of 554 nm. The concentration of samples after dilution ranged from 0-2.72 ×10^−3^ wt% with a coefficient of determination of 0.993.

## Results

### TMS Characterization Results

Based on clinical measurements by Niikawa et al.,^6^ the sensor measurements indicate that the various settings each were exerting pressures on the sensor separately physiologically relevant for tongue strength of young infants. Pressure is shown to vary due to tongue contact location, infant age, and infant birth-weight. Compression setting 1 and rotation setting 2 (C1R2) and compression setting 2 and rotation setting 1 (C2R1) may mimic the tongue pressure exerted at the apical portion of the tongue during breastfeeding, reported clinically as 108-222 KPa.^6^ Compression setting 2 and rotation setting 2 (C2R2) may mimic that of the posterior portion of the tongue since this as has been measured by Niikawa et al.^6^ to range from 231-294 KPa. Compression setting 1 and rotation 1 (C1R1) may mimic the pressures of the apical portion of the tongue by young low birth-weight infants reported as 49.7-222 KPa for a single infant.

### Tablet Characterization Results

The physical characterization data for the tablets, used by Scheuerle et al.^16^ previously, typical for rapidly disintegrating tablets of this formulation, are summarized in Table 2.

### Experimental TMS Results

**Figure 4:**
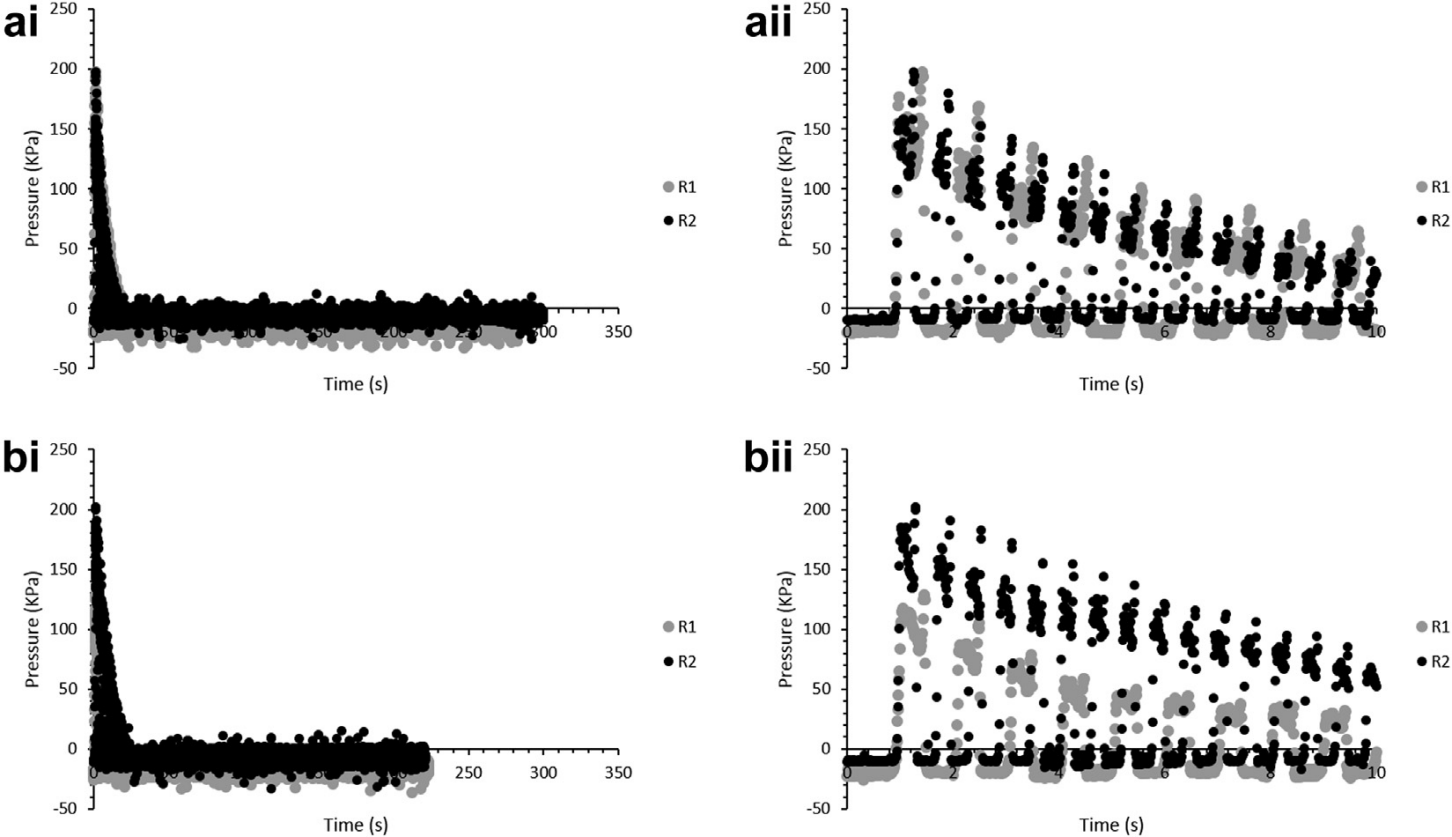
Pressure measurements for the median of 3 trials for compression setting 1 (a) and compression setting 2 (b) for the duration of each trial (i) and for the first 10 s of each trial (ii); graphed with ±1 s accuracy.

**Figure 5:**
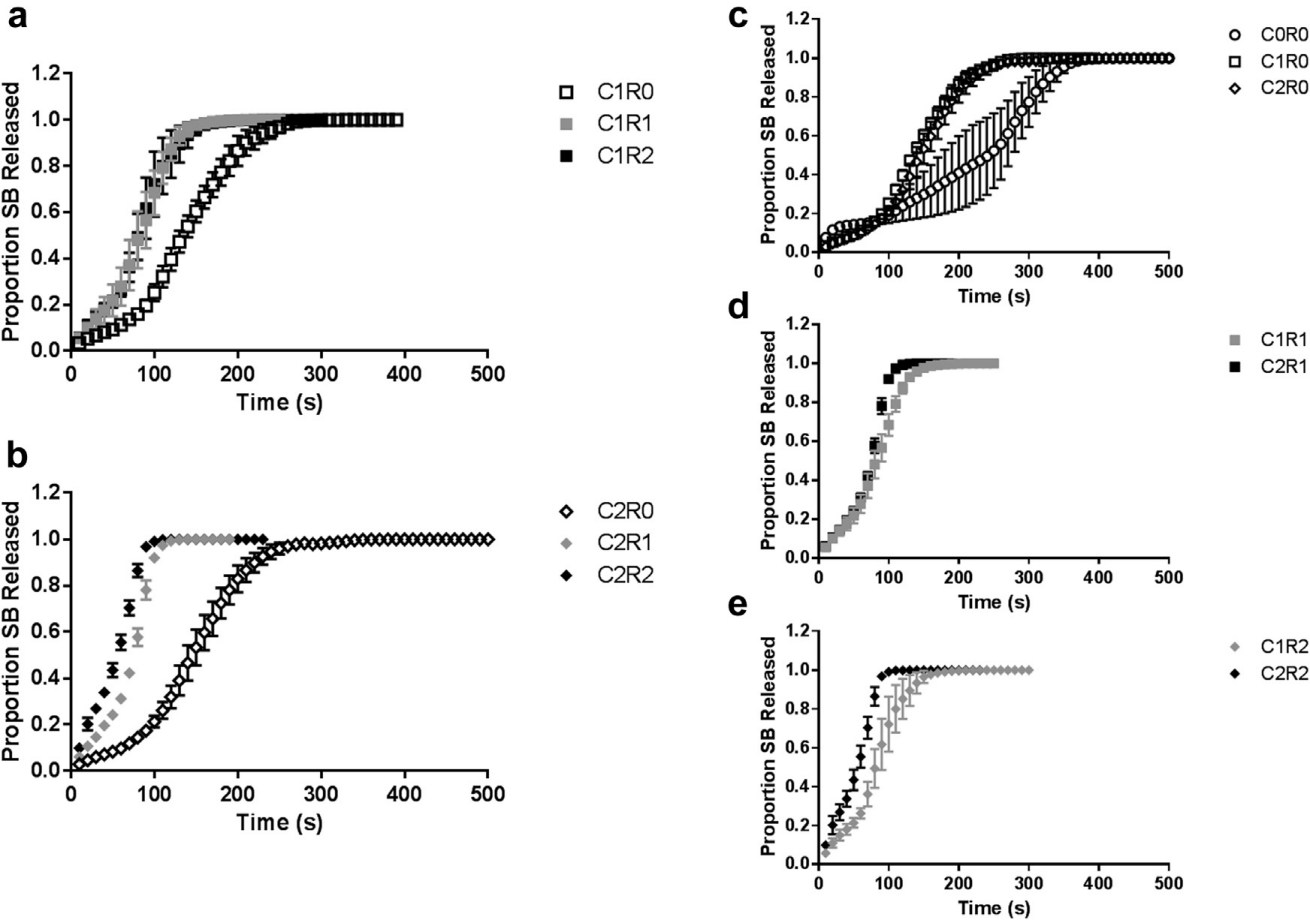
Release profiles for SB for compression setting 1 (a); compression setting 2 (b); and rotational rate 1 (c), 2 (d), and 3 (e); n = 3; graphed with ±10 s accuracy; error bars represent standard error.

Representative pressure profiles during the breastfeeding simulation experiments at C1 and C2, for R1 and R2, measured by the pressure sensor are shown in Figure 4. Release profiles of SB for these trials, and for experiments at no rotation (R0) as well as those at no compression, are shown in Figure 5, grouped by compression setting, and grouped by rotational setting.

Based on the pressure profiles, it is clear that the pressure spikes associated with TM contact during each rotation with the tablet-containing tubing decrease in magnitude over time. This could be attributed to the softening on wetting of the tablet due to the presence of the flowing fluid. This can also be attributed to the loss of tablet into the flowing fluid over time, which decreases the material through which pressure can be propagated to the sensor.

A table of the peak pressures for C1R1, C1R2, C2R1, and C2R2 is summarized in Table 3. In the case of C1, the presence of the water increases the peak pressure for the SB tablet compared to the PEEK tablet, possibly due to the presence of water flowing through the tubing, which initially accumulates on the tablet until partial tablet disintegration. The lower peak pressures for C2 for the SB tablet compared to the PEEK tablet may be due to tablet softening which decreases the propagation of pressure to the sensor.

To compare whether the rotational rate, analogous to the rate of tongue motion associated with infant suction rate, was significant, the release profiles of SB for C1 at R0, R1, R2 were compared, as were those for C2, using 2-way ANOVA with matching (α = 0.05). Rotational rate was found to be significant for both compression settings with *p* = 0.0013 and *p* < 0.0001, respectively. To determine whether the results indicated a difference between rotational rate versus only dependence on the presence or absence of rotation, 2-way ANOVA with matching for each compression setting data set was performed comparing just R1 and R2. In this case, rotational rate was found not to be significant for C1 with *p* = 0.9888, but still to be significant for C2 with *p* = 0.0074.

To analyze the impact of compression setting, analogous to infant tongue strength, 2-way ANOVA with matching was separately performed on the release profiles of SB for each rotational setting at varying compression settings. For no rotation, and for the slowest rotation rate, compression was not found to be a significant factor, with *p* = 0.1556 and *p* = 0.1771, respectively, though for R2, compression setting was found to be significant with *p* = 0.0162.

## Discussion

An infant TMS for simulating infant tongue peristalsis was developed. The system was used to characterize the impact that infant tongue movement during breastfeeding could have on disintegration and dye release of a rapidly disintegrating tablet. This characterization is useful in screening dosage forms to be used in an NSDS, a drug delivery device under development for use during breastfeeding.

Tongue peristalsis during breastfeeding could potentially alter milk flow through the NSDS, as well as contribute mechanical forces to a therapeutic tablet loaded in the device, increasing its rate of disintegration and drug release. The impact of changes in magnitude of compression exerted on a tablet and tongue rotational rate were studied using the TMS and model tablets containing SB. There was a lack of significant difference for the release profiles of SB at different rotational rates for the compression setting C1, whereas for the higher setting C2, the measured pressures were more different from one another. This may be because the flow of fluid is a more significant contributor to tablet disintegration and dissolution under low pressure conditions. At each rotational setting, compression as a variable was compared and found not to be significant for no rotation nor the slowest rotation. Though at the fast rotation R2, compression setting was found to be significant although the measured pressures were similar in these conditions, indicating that pressure is not the only variable affecting the model compound release.

The fastest SB release occurred with the combination of the highest compression and rotation settings. This could be due to the combination of mechanical stress on the tablet under these conditions leading to faster disintegration, and in turn dye release. It could also be due to the efficacy of these settings for forcing the softened disintegrated tablet material through the system to the fraction collector. This could be analogous to how the tongue could both contribute mechanical stress to a tablet in the NSDS during clinical use, as well as for how the tongue motion could extrude disintegrated tablet material out of the NSDS facilitated by the flow of milk.

In the context of the NSDS, these results indicate that for infants which apply more pressure during suckling, potentially due to increased age, suction frequency has a stronger effect on API release rate. For stronger infants, suction rates nearing those in nonnutritive breastfeeding could lead to faster tablet disintegration, but that is assuming sufficient milk flow as was present in this study, which typically is low during this type of feeding. Therefore, this effect could be counteracted in practice by the faster flow rates of milk typical during nutritive feeding^1^ which could increase tablet disintegration. For very young infants, or low birthweight infants, although compression resulting from breastfeeding could increase API release compared to no pressure, the breastfeeding tongue motion rate differences may not be as significant.

To take advantage of the increased drug release possible at higher pressures at fast suction rates, applied tongue pressure could be maximized by strategic dosage form placement in the tip of the device and by ensuring the device sits on the posterior portion of the tongue in the mouth. Placement of the tip of the device containing the tablet on this stronger, posterior portion of the tongue^15^ is expected on account of natural breast placement in the mouth with the nipple in the pharyngeal space, a region where the tongue is shown to exert higher pressures on the nipple.^6^ Future characterization and optimization of the NSDS should consider the target infant population's tongue motions during breastfeeding to ensure appropriate dosage of active pharmaceutical ingredients during use.

Future studies could vary the flow rate of liquid used in the TMS to study the combined effects of various flow rates with various compression and tongue rotation frequencies, to study the feasibility of using the device during various phases of breastfeeding. Future studies could also use human milk as the test medium. Additional studies using other compression levels and suction rates could be performed.

The TMS could be further developed to include additional sensors so that the pressure on other regions of the tablet could be performed. The current system approximates the pressure exerted on the tablet because losses such as that due to dissipation through the tubing occur. Future iterations of the system could measure the dissipation effects due to the tubing to more accurately assess real pressure exerted on the tablet or could include a modified design change which places the sensor against the tablet.

A version of a TMS could be integrated into the breastfeeding simulation apparatus used by Gerrard et al.,^7^ to characterize drug release from the NSDS under physiologically relevant suction and tongue peristaltic conditions. Furthermore, the TMS could also be used to characterize the impact of tongue peristalsis on other products, such as infant pacifiers or artificial nipples.

## Figures and Tables

**Table 1:** Pressure Characterization of Tongue Mimic System Settings

Compression Setting	Rotational Rate (Rotation/min)	*n*	Average Peak Pressure (KPa)^a^	*n*	Potential Clinical Analogue Conditions to the Measured Pressures^b^
Setting 1 (C1)	(R1) 58.5 ± 0.3	18	51 ± 2	3	Apical tongue region of a young low birthweight infant
Setting 1 (C1)	(R2) 105.4 ± 0.5	18	105 ± 6	3	Apical tongue region
Setting 2 (C2)	(R1) 58.5 ± 0.3	24	153 ± 7	3	Apical tongue region
Setting 2 (C2)	(R2) 105.4 ± 0.5	24	260 ± 18	3	Posterior tongue region

a Per rotation based on PEEK tablet peak pressure results for the first 10 rotations of 3 trials.

b Based on clinical data reported by Niikawa et al., 2012.^6^

**Table 2:** Laboratory-Manufactured Tablet Details (Scheuerle et al., 2015^16^)

Composition				
Chemical	Role	w/w	Grade	Manufacturer
Sulforhodamine B (Sigma)	Model compound	2.6	75% Purity	Sigma-Aldrich, Dorset, UK
Lactose (SuperTab 14SD)	Filler	91.4	Ph. Eur	DFE Pharma, Goch, Germany
Sodium starch glycolate (Explotab CLV)	Superdisintegrant	3.0	Typ (A) Ph. Eur	Mendell GmbH, Volklingen, Germany
Croscarmellose sodium (Ac-Di-Sol)	Superdisintegrant	2.0	Ph. Eur	FMC Biopolymer, Girvan, UK
Magnesium stearate	Lubricant	1.0	Technical Grade	Sigma-Aldrich, Dorset, UK

Characterization	
Characteristic	Average ± standard deviation
Height (mm)^a^	4.52 ± 0.08
Width (mm)^a^	5.98 ± 0.02
Diameter (mm)^a^	8.088 ± 0.004
Weight (mg)^a^	330 ± 1
Hardness (*N*)^a^	39 ± 3
Disintegration time (s)^b^	30 ± 2

a *n* = 10.

b *n* = 6.

**Table 3:** Peak Pressure Results of Tongue Mimic System Experiment

Compression Setting	Rotational Rate (Rotation/min)	*n*	Average Peak Pressure (KPa)^a^	*n*
Setting 1 (C1)	(R1) 58.5 ± 0.3	18	188 ± 36	3
Setting 1 (C1)	(R2) 105.4 ± 0.5	18	197 ± 19	3
Setting 2 (C2)	(R1) 58.5 ± 0.3	24	134 ± 12	3
Setting 2 (C2)	(R2) 105.4 ± 0.5	24	188 ± 14	3

a For each trial using SB tablets.
